# Perceptions and experiences of men on antiretroviral treatment lost to follow-up in Govan Mbeki, Mpumalanga

**DOI:** 10.4102/sajhivmed.v27i1.1730

**Published:** 2026-06-03

**Authors:** Sbongile C. Jiane, Mahlatse L. Moropeng, Ndumiso Tshuma

**Affiliations:** 1 School of Health Systems and Public Health, Faculty of Health Sciences, University of Pretoria, Pretoria, South Africa; 2 Best Health Solutions, Johannesburg, South Africa

**Keywords:** perceptions, experiences, lost to follow-up, men, antiretroviral treatment, retention in care

## Abstract

**Background:**

Retention on antiretroviral treatment (ART) is essential for achieving viral suppression and preventing HIV transmission. However, men living with HIV in sub-Saharan Africa are disproportionately lost to follow-up (LTFU), posing significant public health challenges.

**Objectives:**

To explore the perceptions, experiences and barriers to ART adherence among men LTFU on ART treatment at three primary healthcare (PHC) facilities in the Govan Mbeki sub-district, Mpumalanga.

**Method:**

Qualitative, descriptive, and explorative contextual research methodologies were used to collect data among 21 LTFU men from three PHC facilities in the Govan Mbeki sub-district. Participants were selected using heterogenous purposive sampling, and the sample size was determined by data saturation. Semi-structured, in-depth interviews were conducted from May 2024 to July 2024. The data were thematically analysed using NVivo 14.

**Results:**

The study identified key factors affecting ART adherence among men, including stigma, work-related barriers, long clinic waiting times, and medication side effects. Economic pressures, poor service delivery, and negative attitudes from healthcare staff further contributed to ART default. Suggested improvements include extended service hours, male-friendly environments, and better communication with healthcare providers.

**Conclusion:**

This study underscores the complex interplay of barriers driving ART disengagement among men and highlights the need for multi-pronged strategies to improve retention. Addressing these barriers is critical for achieving equitable HIV care outcomes and advancing progress toward the Joint United Nations Programme on HIV/AIDS (UNAIDS) 95-95-95 targets.

**What this study adds:** The study provides insight into the barriers affecting men’s retention in HIV care. It highlights the need for gender response to improve treatment continuity and support achievement of the UNAIDS 95-95-95 targets.

## Introduction

While there is currently no known cure for HIV, antiretroviral treatment (ART) has been the primary treatment since the late 1980s.^1^ HIV has claimed 40.4 million lives and continues to be a global health issue with ongoing transmission in all countries.^2^ Adherence to ART remains a cornerstone of global efforts to combat the HIV/AIDS epidemic.^3,4,5^ Effective ART not only improves health outcomes for individuals living with HIV by suppressing viral load but also plays a critical role in reducing HIV transmission.^6,7^ Globally, men are less likely than women to access HIV testing, treatment, and care, and this results in disproportionate HIV-related mortality rates amongst men.^8^ Retention in HIV care is still a significant goal for HIV services globally, yet default rates continue to vary from 32.7% in America and 12.1% in Europe to 39.4% to 79.4% in Africa.^9^ Achieving and sustaining high levels of adherence poses significant challenges, particularly in sub-Saharan Africa, which bears the highest burden of the epidemic globally.^10,11^

In South Africa, an estimated 7.7 million adults aged 15 years and older were living with HIV in 2023, of which 2.6 million were adult men living with HIV compared to 4.9 million women.^12^ A significant gender disparity exists in terms of HIV testing and treatment; while 96% of women with HIV were aware of their status, only 94% of men had undergone testing.^12^ Moreover, women had better access to ART, with 81% receiving treatment compared to only 71% of men.^13^ Multiple studies have consistently shown that men experience a higher rate of attrition from HIV care programmes compared to women in South Africa.^13,14,15^ Despite having the largest ART programme in the world, men in South Africa continue to experience disproportionately higher rates of disengagement from care and HIV-related mortality compared to women, revealing that men are less likely than women to initiate HIV care, adhere to treatment, and remain engaged in lifelong care.^16,17^ This disparity is attributed to a myriad of factors, including societal norms of masculinity, economic pressures, healthcare system inefficiencies, and the pervasive stigma surrounding HIV.^18^ Loss to follow-up among men in ART programmes not only undermines individual health outcomes but also poses a significant public health threat by increasing the risk of virological failure and HIV transmission.^19,20^ Addressing those lost to follow-up (LTFU) is essential for achieving the Joint United Nations Programme on HIV/AIDS (UNAIDS) 95-95-95 targets, which aim to end the AIDS epidemic as a public health threat by 2030.^21^ South Africa aims to maintain UNAIDS 95-95-95 targets by implementing quality healthcare, universal test and treatment policies, and long-term optimal ART for viral suppression and reduced mortality.^22^ South Africa is currently at 95-77-71 on UNAIDS 95-95-95 targets.^12^

Despite the critical importance of men in HIV care, there is limited understanding of the nuanced barriers that drive disengagement among this demographic, particularly in resource-constrained settings.^23^ Existing literature has often focused on generalised population data, overlooking the unique socio-cultural and structural factors that shape men’s experiences with ART.^24^ To bridge this gap, qualitative research is indispensable, offering rich insights into the lived experiences, perceptions, and barriers faced by men LTFU on ART.

This study explores these issues within the Govan Mbeki sub-district, a high HIV-burden area in Mpumalanga Province, South Africa. The sub-district’s primary healthcare (PHC) facilities report alarmingly high rates of male disengagement from ART programmes, with LTFU rates ranging from 9% to 15% among men initiated on ART between January 2021 and January 2022.^25^

This trend reflects broader national and regional patterns, underscoring the urgent need for targeted interventions.

Using a qualitative, exploratory, research approach, this study investigates the barriers contributing to ART disengagement among men in the Govan Mbeki sub-district. By capturing the voices and experiences of men LTFU, the study aims to provide actionable insights for public health practitioners and policymakers to enhance ART retention strategies. The findings contribute to the growing body of evidence needed to develop gender-responsive healthcare interventions, improve treatment adherence, and ultimately close the gap in HIV care disparities.

## Research methods and design

### Study design

This study employed a qualitative, explorative, descriptive and contextual research design to gain an in-depth understanding of the perceptions and experiences of LTFU men on ART treatment in PHC facilities in the Govan Mbeki sub-district, Mpumalanga.

The qualitative approach was chosen for its ability to provide rich, nuanced insights into participants’ lived experiences and perceptions. The explorative and descriptive components allowed for the identification and documentation of key themes, while the contextual approach ensured that the findings were grounded in the specific sociocultural and healthcare environment of the study setting. This methodological framework was particularly suitable for addressing the complexities of ART adherence and disengagement in resource-limited settings.

### Study setting

This study was conducted in the Govan Mbeki sub-district, one of seven municipalities in the Gert Sibande District, Mpumalanga. Govan Mbeki is recognised as having the fifth largest population in the Mpumalanga province, accounting for approximately 30% of the Gert Sibande district population.^26^ The area is predominantly urban, with pockets of socioeconomic disadvantage, and is characterised by high HIV burden. The study involved three of the 12 PHC facilities in the Govan Mbeki sub-district. These three were chosen based on their high rate of ART initiation and subsequent LTFU among men. LTFU rates in men ranged from 9% to 15% among men initiated on ART between January 2021 and January 2022.^25^

### Study population and sampling

The study used heterogenous, purposive sampling to gather perceptions from men of different ages, occupations, educational levels, perceptions, and experiences, to provide a significant variable in understanding social and health -related behaviours within the study population.

The study population comprised adult men aged 18 years and older who were diagnosed with HIV and initiated on ART at the selected facilities between January 2021 and January 2022, but had not collected their ART for at least 90 days after their last scheduled follow-up visit. This population was targeted because of its critical relevance to understanding ART disengagement. LTFU was defined as being at least 90 days after their last scheduled appointment date for the treatment follow-up visit using the Tier.Net electronic register.^27^ Facility data capturers utilised the Tier.Net electronic register to identify potential participants meeting the LTFU criteria. Lists of eligible men were generated, and routine tracing mechanisms, including physical outreach by Ward-Based Outreach Teams and Coach Impilo, a reimagined peer navigator for men, were used to trace potential participants and inform them about the research topic and purpose of the study.

A total of 21 participants were recruited, with the sample size determined by data saturation, namely the point at which no new themes or insights emerged during data collection. Efforts were made to ensure variability in participants’ age, educational background, employment status, and marital status, to capture diverse perspectives.^28^

### Data collection

Data were collected through semi-structured, in-depth, face-to-face interviews conducted between May 2024 and July 2024. An interview guide was developed to explore the following areas: participants’ experiences with ART, perceptions of treatment and healthcare services, and barriers to adherence. The guide consisted of open-ended questions, allowing participants to express their views freely while enabling the researcher to probe for additional details.^29^ Interviews were conducted in English, with translation into IsiZulu for participants who were more comfortable in their native language. Each interview lasted 20–45 min and was conducted in a private room at the respective healthcare facility to ensure confidentiality and comfort. Field notes were taken to capture non-verbal cues and contextual details, and a digital voice recorder was used to record the sessions with participants’ consent.

To establish rapport, the researcher began with non-directive, open-ended questions and gradually transitioned to more focused topics. Participants were informed about the purpose of the study, and written informed consent was obtained before interviews commenced. Participants were not compensated, as the study only involved interviews and posed no harm.

### Data analysis

Data were analysed using an inductive reflexive, step-by-step thematic analysis approach, following the guidelines proposed by Braun and Clarke. The process began with familiarisation, where interviews were transcribed verbatim, and the transcripts were read repeatedly alongside listening to audio recordings to ensure accuracy and immersion in the data.^30^ Initial codes were then generated by identifying key phrases and patterns within the data and systematically applying these codes to relevant data points^30^ using NVivo 14,^31^ a qualitative data analysis software.

Next, themes were identified by grouping related codes based on their similarities and relevance to the research questions. Relationships between themes were explored to construct a coherent narrative. The themes were then iteratively reviewed and refined to ensure alignment with the data set and participants’ accounts, merging similar themes and discarding those that did not contribute to the overall analysis. Each theme was defined and named to encapsulate its essence, with detailed descriptions and illustrative quotes to support them.^30^

Finally, the findings were synthesised into a cohesive report that integrated direct quotes to substantiate the key points and provide a voice to participants’ experiences. This rigorous and systematic analytic process ensured the credibility and robustness of the findings, offering a thorough understanding of the study’s research questions.

### Ethical considerations

Ethical approval was obtained from the University of Pretoria, Faculty of Health Sciences Research Ethics Committee (reference number: 48/2024) and Mpumalanga Provincial Health Research Ethics Committee (reference number: MP_202404_005) and Gert Sibande Health District and facilities. Participation was voluntary. Participants were clearly informed that they could withdraw at any time or choose not to answer specific questions if they felt uncomfortable. All transcripts’ notes and voice recordings were encrypted for security, and access was limited to the researcher and supervisors. Participants’ data were protected using codes instead of their actual names to ensure confidentiality and privacy of personal data. Permission for voice recording was obtained before the interviews.

## Results

The results shown in Table 1 indicate limited representation from younger age groups. The data also reveal that while a majority have at least completed high school, a smaller segment pursued education beyond the secondary level. The relatively high unemployment rate reflects the socio-economic challenges facing the population under study, which may affect health access and service utilisation. The homogeneity in religious affiliation may reflect broader societal trends within the region and may influence certain health-seeking behaviours or cultural practices related to health. These demographic results provide a foundational understanding of the population, which will be further explored in the context of the study’s specific health outcomes and behaviours.

**TABLE 1:** Socio-demographic profile of participants (*N* = 21).

Demographic factors	Sub-category	*n*	%
Age (years)	18–25	1	4.8
	26–30	2	9.5
	30–35	4	19.0
	36–40	5	23.8
	41–49	7	33.3
	50–54	2	9.5
Educational level	Below matric	6	28.6
	Matric	11	52.4
	Post matric	4	19.0
Occupation	Employed	12	57.1
	Unemployed	9	42.9
Marital status	Single	14	66.7
	Married	6	28.6
	Divorced	1	4.8
Religion	Christian	21	100.0

The results highlight several interrelated barriers contributing to ART non-adherence among men in the Govan Mbeki sub-district. Thematic analysis revealed the following three themes and their sub-themes: Theme 1. Barriers to ART adherence, consisting of four sub-themes; Theme 2. Healthcare system inefficiencies, with three sub-themes; and Theme 3. Facilitators of ART adherence, with two sub-themes. The data underscore the complex interplay of social, economic, and systemic factors that drive loss to follow-up among men.

### Theme 1: Barriers to antiretroviral treatment adherence

Several participants reported how ART negatively impacted on their daily routines and work, work-related barriers to accessing ART, stigma, and discrimination associated with HIV/AIDS; some also reported experiencing medication side-effects, and described how they used coping strategies to overcome barriers to ART adherence.

#### Stigma and discrimination

Stigma and discrimination emerged as critical barriers to ART adherence, both at the community level and within participants’ relationships. Participants described a profound fear of being labelled as HIV-positive, leading to concealment of their treatment status and, in some cases, complete avoidance of healthcare facilities. Men would rather risk non-adherence than expose themselves to potential ridicule. One participant articulated this sense of fear and the lengths to which he would go to avoid being discovered taking ART:

‘Sister, you know how people gossip about you when they know that you have HIV. Whenever I need to drink medication, I must run to the toilet, and on the days where I do not have the opportunity to go to the toilet, I will miss my medication dose.’ (54 years, basic education, employed, married)

The stigma was not only external but also internalised, with some men expressing feelings of shame and worthlessness. For some, even familial relationships, which might have served as a source of support, became sources of discrimination:

‘I felt discriminated against by my own family, so I no longer see a reason to continue with the treatment. I do not care what happens to me anymore.’ (44 years, tertiary education, employed, married)

#### Work-related challenges and economic pressure

Work-related demands, particularly for men in occupations requiring frequent travel or long hours, were another significant barrier to ART adherence. Many participants described difficulties in aligning their treatment schedules with their job requirements. A participant working as a long-distance truck driver explained:

‘I am a long-distance truck driver, and I can’t always come to the clinic because I’m always on the road. When I send my wife to collect my medication, the nurses refuse to give it to her.’ (52 years, basic education, employed, married)

Another participant echoed similar concerns, emphasising the unpredictability of his work schedule:

‘We are always away from home, sometimes for months. It’s difficult to get medication in those countries because they require a transfer letter, and we don’t always know when we’ll be leaving.’ (48 years, basic education, employed, single)

Interestingly, some participants reported that their employers were accommodating and allowed time off for clinic visits. These instances of support highlight the potential for workplace policies to mitigate this barrier; others, however, reported that economic hardships forced them to prioritise job hunting over clinic visits. For those unemployed or underemployed, securing a livelihood took precedence over attending health appointments, especially when missing work meant potential job loss or financial instability. This economic pressure led to irregular clinic visits, contributing to challenges in adhering to ART:

‘I am not working and have a young child to care for, so I do some piece jobs. That’s why I am not coming because the day spent at the clinic waiting the whole day, I used it to look for piece jobs and make money.’ (34 years, matric, unemployed, single)

#### Medication-related barriers

Medication-related side effects were another common concern. Participants reported symptoms such as dizziness and fatigue, which interfered with their daily activities and work responsibilities and sometimes led them to skip doses or stop treatment altogether. One participant recounted:

‘The pill I take at night always makes me feel dizzy and sleepy. I had difficulty waking up and was always late for work. I stopped taking them, but I heard there’s a new pill now that I haven’t tried.’ (46 years, matric, employed, divorced)

#### Coping strategies and alternative medicines

Some men reported using various coping strategies to overcome barriers to ART adherence; some resorted to sharing antiretroviral medications with friends or family members because of various barriers to accessing their prescriptions. This practice was reported as a temporary solution during periods when they ran out of medication, travel constraints, or financial difficulties that prevented clinic visits. However, this behaviour disrupted the continuity of their treatment regimens and increased the risk of drug resistance:

‘I promise you; I still drink my ART because one of my gents shared his with me. My sister, who wants to die of HIV.’ (40 years, matric, unemployed, single)

Others mentioned switching healthcare facilities as an alternative to access ART. This was often because of dissatisfaction with services, poor treatment from staff, or the need for greater convenience. Switching clinics sometimes allowed them to avoid long waiting times or negative staff attitudes at their primary facility, which is why they appear as if they are LTFU. However, they are still in care at other facilities, but they did not take referral letters:

‘I have not stopped drinking my pills, I now collect them at Trichardt clinic because the queue is fast there.’ (54 years, basic education, employed, married)

Some men explored alternative treatments alongside or instead of ART. These alternatives ranged from traditional medicine to over-the-counter vitamins, motivated by cultural beliefs or a desire to boost their immunity. However, this often led to non-adherence to prescribed ART regimens and worsened health outcomes:

‘Yes, I am also drinking traditional medication to boost my immune system; as a man, you need to cleanse your system so that you are strong.’ (34 years, basic education, unemployed, single)

Together, these strategies reflect how men navigated system barriers to ART access, balancing cultural practices, convenience and necessity, although often at the expense of consistent adherence; while these practices served as temporary coping mechanisms, they disrupted treatment continuity and increased risk of drug resistance.

### Theme 2: Healthcare system inefficiencies

Healthcare system challenges, particularly long waiting times and negative staff attitudes, were frequently cited as reasons for disengagement.

#### Long waiting times

Many participants expressed frustration with the time required to access care, which often conflicted with their work and family responsibilities. One participant shared:

‘You spend the whole day at the clinic waiting for your medication. I usually ask to come late to work to visit the clinic, but if I miss a full day, I won’t be paid. It’s a lot of money, so I decided not to come anymore.’ (47 years, matric, employed, single)

#### Healthcare workers’ attitudes

Healthcare workers’ attitudes also emerged as a significant barrier. Participants described feeling judged or scolded during their clinic visits, which discouraged them from returning:

‘The service we get from nurses is not good. They are cheeky and treat us like children. That’s why I stopped coming to the clinic.’ (43 years, tertiary education, employed, married)

Interestingly, a minority of participants described positive experiences with healthcare staff, indicating that not all interactions were negative. These accounts suggest variability in service quality across different facilities.

#### Support services for men on antiretroviral therapy

Most men were unaware of any male-specific services at the clinics. Men expressed that they were not informed about male support that could have helped them navigate the challenges of living with HIV and adhering to ART.

### Theme 3: Facilitators of antiretroviral treatment adherence

Despite the challenges, some men expressed acceptance of their HIV status and a solid commitment to adhering to ART. This commitment was often linked to a desire for better health, survival, and the well-being of their families. These individuals viewed ART as essential to managing their condition and were determined to stay on treatment:

‘You must accept that responsibility, and it must also be registered in your mind. Ya, although it is difficult, you must learn to live with it.’ (41 years, tertiary education, employed, married)

Most men reported significant improvements in their health after initiating ART. These positive changes, such as increased energy, weight gain, and fewer illnesses, reinforced their commitment to treatment. Improved health outcomes served as a motivating factor for continued adherence and clinic visits:

‘When I started hearing that I am HIV positive, I was angry, and I was so sick. Also, having TB [*tuberculosis*] treatment helped me a lot.’ (38 years, primary education, unemployed, single)

#### Suggested recommendations for improvements

Participants provided several suggestions for improving ART services for men, including male-friendly environments, better nurse attitudes, shorter waiting times, flexible service hours, and designated staff for missed appointments.

Most men proposed the availability of weekend clinic hours as a crucial improvement for ART services. Weekend access would provide a convenient option for those unable to attend during regular weekday hours because of work or other commitments. The availability of evening clinic hours was also seen as critical for employed men to provide a flexible option for those unable to attend during regular working hours, making it easier to maintain treatment without compromising their employment:

‘Yes, having more male nurses and the clinic give us our treatment during that night as it is open 24 hours, it will be easy for us. At work, they are telling us that we are going late on the day that we are going the next day, it will be easy for us to rush to the clinic when we knock off to explain to nurses and ask for treatment.’ (48 years, basic education, employed, single)

Men emphasised the importance of male-friendly environments at clinics. They appreciated facilities that provided privacy, understanding staff, and a supportive atmosphere tailored to their needs. Such environments made them feel more comfortable seeking care and increased their willingness to engage with health services:

‘Yes, my sister, as a man, I would be happy to be treated by other men so that we can talk men to men, you know, as a man, don’t feel comfortable pouring my heart into women’s nurses.’ (49 years, matric, married, employed)

Participants suggested designating specific clinic staff to follow up with patients who miss appointments and fast-tracking proactive contacts via phone calls or home visits. This approach addresses treatment default challenges by providing targeted interventions for those at risk of being LTFU:

‘Yes, but by that, I don’t mean we should not be responsible for our health can we have someone who will assist us if we miss our appointment if we come back just go straight to her without any fear of being shouted at.’ (41 years, tertiary education, employed, married)

To address long clinic waiting times, men suggested strategies such as having more staff on duty during peak hours, allowing access to the clinic after hours, and referring stable patients to pharmacies. Reducing waiting times would encourage more consistent clinic visits and improve patient satisfaction:

‘Yes, improve their waiting time, we wait for long.’ (40 years, matric, unemployed, single)

Barriers to ART adherence, healthcare system inefficiencies, and facilitators of ART adherence emerged as dominant themes, that show a complex mix of barriers that contribute to ART non-adherence among men LTFU; there were also examples of resilience and support that highlighted opportunities for targeted interventions. Understanding these varied experiences is critical for designing effective, context-specific strategies to improve ART retention in this population.

## Discussion

This study highlights the multifaceted barriers contributing to ART non-adherence among men LTFU in the Govan Mbeki sub-district, and emphasises the interplay between societal norms, healthcare system inefficiencies, and individual challenges. The findings align with existing literature while offering unique insights into the lived experiences of men disengaged from HIV care.

Stigma remains a persistent barrier, with participants describing the pervasive fear of discrimination and its impact on their ability to adhere to ART. Internalised stigma, compounded by societal perceptions of masculinity that discourage vulnerability and health-seeking behaviour, played a significant role in disengagement. These findings are consistent with global evidence highlighting the detrimental effect of stigma on ART retention.^19,21^

However, this study reveals a nuanced layer of how stigma manifests in everyday scenarios, such as concealing medication use or avoiding healthcare facilities altogether. Addressing stigma requires interventions that not only challenge societal attitudes but also empower individuals. By incorporating elements of human-centred design, interventions can be co-created with the community to ensure cultural relevance and acceptability. Peer-led stigma-reduction campaigns, informed by men’s lived experiences, have proven effective in fostering supportive environments.

Economic instability and work-related challenges emerged as significant barriers, particularly among participants in mobile or informal employment. Men reported prioritising income over healthcare, with inflexible work schedules further limiting their ability to attend clinic appointments. These findings resonate with studies across sub-Saharan Africa, where economic pressures often hinder ART adherence.^11,32^

The study also highlights the unique challenges faced by men in mobile professions, such as truck drivers, who struggle with continuity of care because of regional mobility and administrative barriers. This underscores the need for portable ART services and policies facilitating seamless transitions between facilities. Digital health interventions, such as telemedicine and app-based reminders, can be tailored to meet the needs of this group, potentially improving adherence outcomes.

Coping strategies, such as sharing medication with others or switching healthcare facilities, were used as an alternative method when they were unable to collect their treatment. The finding of this study ties well with a previous study, which reveals that participants who like to transfer clinics within the same catchment area reflect worse retention and clinical outcomes.^33^ Long waiting times and negative attitudes from healthcare workers were repeatedly cited as drivers of disengagement. Participants described feeling disrespected or judged during clinic visits, echoing findings from other studies that link poor patient-provider interactions to reduced retention in care.^10,32^

Improving healthcare service delivery requires systemic changes, including training for healthcare workers to provide empathetic, non-stigmatising care. Facility-level reforms, such as streamlining ART delivery processes and extending clinic hours, could address logistical barriers while fostering a more inclusive healthcare environment. As highlighted by this study, even small changes, such as respectful interactions and shorter wait times, can significantly impact men’s willingness to remain in care.

Participants reported medication side effects, including dizziness and fatigue, as significant barriers to adherence. These findings align with global studies documenting the impact of adverse drug effects on patient retention.^9,19^ While newer ART regimens with fewer side effects are increasingly available, this underscores the importance of comprehensive patient education during treatment initiation and transition.

Some participants expressed a preference for traditional medicine alongside ART, reflecting the importance of cultural context in treatment adherence. Integrating traditional healers into HIV care programmes, as seen in other African settings, could foster greater acceptance and adherence among patients with strong cultural ties to traditional practices.

While most participants reported negative experiences, a few described supportive workplace policies and positive interactions with healthcare workers. These outliers suggest that some facilities or employers have already implemented effective practices, providing a blueprint for broader implementation. Identifying and scaling these successes could serve as a cost-effective strategy for improving ART retention across the sub-district.

Human-centred design principles could play a pivotal role here by engaging men and other stakeholders in co-creating tailored solutions. For instance, identifying specific preferences for service delivery or designing male-friendly healthcare spaces could address common barriers while improving user satisfaction.

The findings highlight the need for multi-level interventions addressing the structural, social, and systemic barriers to ART adherence. Policies must prioritise stigma reduction, flexible service delivery, and economic support to accommodate the unique needs of men. Gender-responsive strategies, such as male-targeted health campaigns and workplace-based ART delivery, are essential to dismantle the societal norms that deter men from seeking care.

Innovative approaches, such as integrating digital health tools into ART programmes, offer additional potential for addressing logistical barriers. For example, automated reminders and telehealth consultations could improve accessibility and continuity of care, particularly for men in mobile or informal employment.

### Strengths and limitations

This study provides rich qualitative insights into ART disengagement among men in a resource-constrained setting. However, its focus on a single sub-district may limit generalisability. Future research should explore these themes across diverse settings and populations to inform scalable, context-specific interventions.

## Conclusion

This study provides critical insights into the multifaceted barriers to ART adherence among men LTFU in the Govan Mbeki sub-district, Mpumalanga. The findings reveal that stigma, economic pressures, healthcare system inefficiencies, and medication-related challenges collectively contribute to disengagement from care. These barriers are further compounded by societal norms of masculinity, which discourage health-seeking behaviour and perpetuate internalised stigma.

The study highlights the urgent need for gender-responsive and context-specific interventions to address these challenges. Stigma-reduction campaigns targeting both community perceptions and individual experiences, combined with workplace policies that accommodate healthcare needs, can mitigate many of the identified barriers. Furthermore, healthcare systems must be strengthened to offer more patient-centred, male-friendly services, including flexible clinic hours, improved patient-provider communication, and streamlined ART delivery processes. Innovations such as telehealth and portable ART services for mobile populations offer additional potential for improving retention.

While the findings are from the Govan Mbeki sub-district, they echo broader patterns observed across sub-Saharan Africa, emphasising the universal relevance of addressing gendered disparities in HIV care. Future research should expand on these findings by exploring ART disengagement among men in diverse settings to inform scalable interventions.

Ultimately, improving ART retention among men is critical for achieving the UNAIDS 95-95-95 targets and advancing global progress toward ending the HIV epidemic. This study underscores the importance of a multi-pronged, inclusive approach that prioritises the unique needs of men while fostering systemic and societal change to support long-term engagement in care.
